# Patterns of Risky Sexual Behaviors and Associated Factors among Youths and Adolescents in Vietnam

**DOI:** 10.3390/ijerph17061903

**Published:** 2020-03-14

**Authors:** Ha Ngoc Do, Diep Ngoc Nguyen, Hoa Quynh Thi Nguyen, Anh Tuan Nguyen, Hiep Duy Nguyen, Thanh Phuong Bui, Thao Bich Thi Vu, Khiet Thanh Le, Dung Tuan Nguyen, Cuong Tat Nguyen, Linh Gia Vu, Giang Thu Vu, Bach Xuan Tran, Carl A. Latkin, Roger C. M. Ho, Cyrus S. H. Ho

**Affiliations:** 1 Youth Research Institute, Ho Chi Minh Communist Youth Union, Hanoi 100000, Vietnam; ngochayri@gmail.com; 2 Institute for Global Health Innovations, Duy Tan University, Da Nang 550000, Vietnam; nguyenngocdiep7@duytan.edu.vn; 3 Faculty of Pharmacy, Duy Tan University, Da Nang 550000, Vietnam; 4 Department of Research on Youth Culture and Lifestyle, Youth Research Institute, Ho Chi Minh Communist Youth Union, Hanoi 100000, Vietnam; hoaquynh1801@gmail.com (H.Q.T.N.); tuananhtwd@gmail.com (A.T.N.); 5 Department of Research on Children’s issues, Youth Research Institute, Ho Chi Minh Communist Youth Union, Hanoi 100000, Vietnam; duyhiepk53xhh@gmail.com (H.D.N.); thanhxhh26@gmail.com (T.P.B.); 6 Department of Research on Youth’s Organisations and Youth Campaign, Youth Research Institute, Ho Chi Minh Communist Youth Union, Hanoi 100000, Vietnam; bichthaoussh@gmail.com (T.B.T.V.); lethanhkhiet2308@gmail.com (K.T.L.); 7 Department of Research on Youth and Legal issues, Youth Research Institute, Ho Chi Minh Communist Youth Union, Hanoi 100000, Vietnam; dungtuannguyen1711@gmail.com; 8 Faculty of Medicine, Duy Tan University, Da Nang 550000, Vietnam; 9 Center of Excellence in Evidence-based Medicine, Nguyen Tat Thanh University, Ho Chi Minh City 700000, Vietnam; linh.coentt@gmail.com (L.G.V.); giang.coentt@gmail.com (G.T.V.); 10 Institute for Preventive Medicine and Public Health, Hanoi Medical University, Hanoi 100000, Vietnam; bach.ipmph@gmail.com; 11 Bloomberg School of Public Health, Johns Hopkins University, Baltimore, MD 21205, USA; carl.latkin@jhu.edu; 12 Department of Psychological Medicine, Yong Loo Lin School of Medicine, National University of Singapore, Singapore 119228, Singapore; pcmrhcm@nus.edu.sg; 13 Institute for Health Innovation and Technology (iHealthtech), National University of Singapore, Singapore 119077, Singapore; 14 Center of Excellence in Behavioral Medicine, Nguyen Tat Thanh University, Ho Chi Minh City 700000, Vietnam; 15 Department of Psychological Medicine, National University Hospital, Singapore 119074, Singapore; cyrushosh@gmail.com

**Keywords:** risky sexual behaviors, adolescents, youths, condom use, alcohol, unintended pregnancy

## Abstract

Youths and adolescents are vulnerable to HIV/STIs from unprotected sex. Promotion of young population’s awareness about risky sexual behaviors is essential to develop contextualized interventions. A cross-sectional study was conducted in five Vietnamese provinces to document current attitudes and practices regarding sexual behaviors among youths. The information on sociodemographic characteristics, substance use, and sexual behaviors was collected via self-reported questionnaires. The factors associated with risky sexual behaviors were identified by the multivariate logistic regression. Among the 1200 participants, 73.5% reported having sex in their lifetime, and 48.1% used condoms at their latest sexual intercourse. Participants in urban areas were more likely not to intend to use condoms and had a higher unintended pregnancy rate than in rural areas. Older age was positively associated with not wanting to use and not using condoms. Substance-using participants were more likely to not use condoms. The participants taking alcohol or other stimulants before sex had a higher likelihood of unintended pregnancy. Respondents’ attitudes and practices regarding sexual behaviors were associated with gender and employment. This study indicated that young population’s awareness in Vietnam is high, however, risky sexual behaviors also remain common. Sex-related educational programs about the consequences of substance use, multiple sex partners, and unprotected sex should be developed.

## 1. Introduction

Risky sexual behaviors among youths and adolescents are a global public health issue [1,2]. Young people experience substantial physical and psychosocial sequelae of the HIV infection [3]. To effectively prevent and control the HIV infection, more researche is needed to study risky behaviors among youths and adolescents in a culturally relevant context in order to fill research gaps [4,5]. The prevalence of young people reporting that they do not use condoms consistently during sexual intercourse has been found to be relatively high, e.g., in 2013, this rate was about 40% in the USA [6]. Having multiple sex partners or concurrent relationships among youths is also common [7,8]. Unprotected sexual practices substantially increase the risk of sexually transmitted infections (STIs) and unintended pregnancies [9,10]. Although condomless sexual activities have decreased in the recent years, it has been insufficient to curb the epidemic of HIV/AIDS and STIs [11].

Extensive research has shown that the demographic, behavioral, and social factors associated with risky sexual behaviors among the youths included being male, younger age, substance use (illicit drug or alcohol use, tobacco), interpersonal influence (peers’ behaviors), and family structure [12,13,14,15]. Previous studies have shown evidence that supportive parent-child relationships may reduce the risk of unprotected sexual behaviors among teens [16,17]. Adolescents and youths in disadvantaged communities, such as rural, mountainous, and other underdeveloped areas, are more likely to have higher sexual risks [17]. Youths who attended school-based programs, such as comprehensive sex- and HIV-education programs, have been found to have less sexual risk taking and lower pregnancy rates [18]. Several programs have been implemented, such as VFOK (Vietnamese Focus on Kids), the gender-based EWA (Exploring the World of Adolescents) program, and EWA+ (EWA plus parental and health provider education) [19] to improve health literacy of adolescents and parents about the associated sexual risks.

Currently, new factors of risky behaviors may be more pervasive, such as the use of newly emerging synthetic drugs (synthetic cathinone, synthetic cannabinoids, ecstasy, ketamine, or methamphetamine) [20,21], or virtual interactive activities (e.g., online sexting [22] or online sexual harassment [23]). These emerging issues may lead to risky behaviors, such as higher probability of unsafe sex, mental health conditions, and drug dependence [22,24,25,26]. The emergence of these problems may lead to the change of sexual behavioral patterns, especially among youths and adolescents [27,28]. These risky behaviors are also influenced by peers [29,30], and the updated information on risk factors is critical.

In Vietnam, two previous national surveys in 2003 and 2008 indicated that the mean age of sexual activity has reduced in both genders [31]. However, we do not know what changes have occurred since 2008. There have been several studies on sexual behaviors among the young population [32,33], but they might not be representative of the whole young population in Vietnam. More recent studies suggest that the proportion of those consistently using condoms in practice is not high [31,34]. Likewise, the Vietnamese youth have a very high abortion rate, and abortions in unregistered medical facilities are alarming [35,36]. These findings suggest that there is a gap between knowledge and risky behaviors among youths in Vietnam. This study was conducted for the purpose of clarifying current awareness, attitudes, and practices regarding sexual behaviors among young Vietnamese people.

## 2. Materials and Methods

### 2.1. Study Settings and Sampling Method

Five provinces in Vietnam, including Hanoi, Cao Bang, Kon Tum, Binh Thuan, and Dong Thap, were chosen to conduct our cross-sectional study. In each province, six communes were purposefully selected to represent urban, rural, and mountainous areas (two communes per area). Participants from rural, urban, and mountainous areas were invited to an interview if they met eligibility criteria: (1) were 16–30 years old, (2) agreed to participate in the study, and (3) had the ability to read and answer the questionnaire. We used a convenience sampling method to recruit participants. A total of 1200 participants enrolled in the study (95.8% of the total 1252 youths who were invited). The Institutional Review Board (IRB) of the Youth Research Institute approved this study’s protocol.

### 2.2. Measurements

First, a questionnaire was sent to 10 youths as a pilot to test the self-administrated questionnaire, and only minor changes were made based on their comments about the language of each item. The revised questionnaire was then used to collect respondents’ information. It took approximately 10 minutes to complete the questionnaire. Participants were provided information about the study and interested individuals before they provided informed consent. Each participant was invited to a private area to ensure confidentiality. Sociodemographic information included gender, living area, occupation, and age. Here are some examples of questions in our questionnaire: 1. Do you want to use a condom when having sex? Choose 1 option only: 1. Yes, 2. No; 2. Have you ever had sex in your lifetime? Choose 1 option only: 1. Yes, 2. No; 3. Have you ever had unintended pregnancy/made someone pregnant? Choose 1 option only: 1. Never, 2. Once, 3. More than 2 times, 4. Do not remember.

Sexual behaviors: We asked respondents about their sexual behaviors, the age at the first sex, number of sex partners, whether they take alcohol or other stimulants before having sex, whether they use condoms for birth control, and whether they had ever had unintended pregnancy or made someone pregnant.

Substance use: Self-reported data were used to identify if participants are alcohol drinkers and/or use alcohol before having sex. Moreover, participants were asked if they smoked (cigarettes, water pipe, shisha, etc.), the age at the first time they smoked. Participants were considered current smokers if they smoked in the last 30 days. Participants were also questioned if they had ever used other substances, like heroin, or other drugs, and a reason for use.

### 2.3. Statistical Analysis

STATA version 14.0 (Stata Corp. LP, College Station, TX, USA) was used to analyze data. The chi-squared test assessed the differences between the two groups of patients who had or never had sex. The logistic regression model with multivariable adjustment was used to find the factors associated with participants’ attitudes and practice of sexual behaviors. A forward stepwise selection strategy was employed to discard non-significant factors in our study. The log-likelihood ratio test’s *p*-value was set at less than 0.2, which was adjusted as the threshold to select a variable. *P*-value < 0.05 was considered statistically significant.

## 3. Results

Table 1 shows that there were no differences between men and women as to the proportion of the individuals who reported ever having sex (*p*-value = 0.88). The proportion of people from 25 to 30 years of age who had ever had sex (49%) approximated the total proportion in the groups of 16–18-year-olds and 19–24 year-olds (51%). 71.1% of the participants were alcohol drinkers, which is significantly high. Out of the three living areas, the urban area was the location that had the highest proportion of the individuals who reported ever having sex (39.3%), followed by the mountainous setting (32.7%); the rural area featured the lowest figures (28%). High school students and undergraduate students were the two groups who had the lowest proportion of the individuals who had ever had sex (9.3% and 16.3%).

Table 2 shows that 67% of the participants want to use condoms when having sex, while only 48.1% used a condom at the latest sexual intercourse. The proportion of people in mountainous areas who want to use condoms is 73.3%, whereas in urban areas, this number was 61.2%. The proportion of people who took alcohol or other stimulants before having sex was relatively high (50.3%). The percentage of individuals wanting to use condoms among the participants who had 1–2 sex partners (65.0%) is significantly higher than among the people who have more than 2 sex partners.

Table 3 shows the factors associated with attitudes and practices of sexual behaviors of the respondents. The participants in urban areas were more likely to not want to use condoms (OR = 1.48, *p*-value < 0.01) and have unintended pregnancy (OR = 1.86, *p*-value < 0.01) than in rural areas. Older age was positively associated with not wanting to use condoms and not using condoms. The people who use substances, such as shisha and heroin, are more likely to report not using condoms (OR = 10.5, *p*-value < 0.01). The participants taking alcohol or other stimulants before having sex had a higher likelihood of unintended pregnancy. Gender and employment were also associated with attitudes and practices of sexual behaviors. The number of sex partners was negatively associated with the want to use condoms (OR = 2.18, *p*-value < 0.01).

## 4. Discussion

Our study contributes information on sexual behaviors among the youth in Vietnam. The findings of this study indicated that the rates of young people who did not want or did not have the intention to use condoms during sexual intercourse were alarming. Moreover, the rate of unwanted pregnancy was likely to increase in the coming years. The factors associated with risky sexual behaviors among Vietnamese youths included gender, living area, educational level, as well as alcohol and drug use.

In this study, we observed that the prevalence of youths ever having sexual intercourse was nearly three-fourths, and 51% among the youths aged 16–24 years. Compared with the previous research, our result was slightly higher than 42.6% to 48% in the USA [37,38,39], or 35% in Kenya [40]. However, although more than two-thirds of our sample had the intention to use condoms when having a sexual intercourse, the rate of condom use at the latest sexual intercourse was only 51.9% in the whole sample, and only 48.1% among those having the intention to use a condom. This finding shows a significant gap between practice and intention towards condom use among Vietnamese youths. Our rates of condom use and intention were significantly lower than those in a previous nationally representative survey in 2008 among youths [31] and other studies in both developed and developing countries [41,42]. The rate of consistent condom used in developing countries (around 17% to 22%) still remains low and has had a tendency to decrease since 2006 [43,44,45,46,47,48]. Several reasons may help to explain this phenomenon. First, discussing condoms is still considered a sensitive subject in Vietnam [49]. Second, there was evidence that higher level of family communication was one of the important associated factors to lower the level of risky behaviors among the youth. The association between parental communication about sex and adolescents’ self-esteem was significantly high, and if this communication is not effective enough, the use of condoms among the youth might decrease [50,51,52]. In addition, youths may think they can efficiently control risks during a sexual intercourse in serious, steady, and safe relationships [53,54,55]. Notably, the prevalence of the young people who had unintended pregnancy or made someone pregnant was nearly 20%. This rate is quite high even when it is compared to the abortion rate in Vietnam (15.7% in 2013) [56], suggesting an urgent need for intensive sexual reproductive health education for youths and adolescents in Vietnam.

Socioeconomic characteristics, including age, gender, education, and living area, were associated with sexual behaviors among the youth, which was consistent with previous findings [57,58]. Women are the most vulnerable group to not using condoms or unintended pregnancy due to some unexpected problems such as forced sex/sexual violence [59] or resistance of their sex partner when having sex [60,61,62]. This seems related to incidences of male sexual violence toward females, and males coercing females into sexual activity. Male power, patriarchal cultures, man’s “natural” sexual urge, sexual jealousy among young men while competing for females were considered as associated factors which led to male-female sexual violence [63,64,65]. In addition, younger people were more likely to have the intention of using condoms compared to older ones. One explanation could be that the young age group was the dependent group in Vietnam because of not having enough financial capacity, and their discernment of the high risk of not using condoms during a sexual intercourse, such as unintended pregnancy, made them proactive in protecting themselves [66]. Moreover, this finding could be an impact of school-based sexual education programs for students. Another possible explanation was that older people might choose other prevention methods (such as pills, injectables, withdrawal, spermicides, periodic abstinence, hormonal methods) [67,68]. Furthermore, we found that condom use rates were lower in urban than in mountainous areas in our study, which might be contributed by the diversity of pills and other modern contraceptives. This contraception use pattern has been observed in other regions [69,70,71,72].

The current study indicated that youths with multiple sex partners (3–5 partners) are inclined to have lower intentions to use condoms. This finding was consistent with previous studies, which indicated that people who had multiple sexual partners were less likely to use condoms to prevent STIs and HIV/AIDS [73,74]. Likewise, people who used stimulants, such as shisha annd heroin, were found to have significantly high rates of not using condoms during a sexual intercourse. Prior research on stimulant users illustrated that they had relatively common risky sexual behaviors [75,76,77,78]. It has been noted in previous studies in various countries, including Vietnam, that there is a link between drug use and unsafe sex [79,80,81,82]. Notably, the lack of knowledge among the youth about health-related consequences of using stimulants (such as shisha, heroin) [83,84,85], including HIV and HCV infection risks, is well documented [82]. 

Interestingly, our results pointed out that the participants who drank alcohol were likely to use condoms. However, the rate of unwanted pregnancy in the group taking alcohol and other stimulants before having sex was significantly higher than among non-drinkers. The reduction of alcohol intake before having sex could be considered as one effective proposal to limit the risks of unprotected sex and unsafe sex [86]. A possible school-based program could be incorporated to help youths understand the association between alcohol and risky sexual behaviors (HIV/STI risks), and other potential negative health consequences [87]. Besides the common knowledge of sexual behaviors, sexual education programs could be specific for each group, such as for young female population, the program might guide them on how to react in case of forced sex and reinforce the need in reproductive health screening. For young males, the programs might include a guideline on correct condom use. Previous research found that young Vietnamese prefer to download smartphone applications for disease prevention [88]. As a result, campaigns on the Internet and social media, and the use of online healthcare providers should be promoted to improve awareness of STIs [89]. Digital sex education is a potential solution for adolescents and youths together with the development of technology [90,91]. Parental communication between parents and their children about risky sexual behaviors should be more frequent in order to enhance the proper awareness of adolescents about safe sex [92]. Parents should also be involved in sex education programs designed for their children to increase the effectiveness of these programs [93]. Sex education policies have been verified to be effective in Sub-Saharan Africa, because these policies increased awareness of the young people about the importance of HIV prevention and reproductive health [94]. In other countries, like Canada and the USA, these policies also showed their effectiveness in providing proper knowledge about human sexuality and avoiding sex-related health issues [95]. Furthermore, medical facilities could intervene by providing advisory services for young clients about hazardous behaviors while and after drinking alcohol [96].

Our study has several limitations. First, as we used the cross-sectional study design, the determination of the causal relationship between independent and dependent variables was partly restrained. Secondly, it might not be representative of all of the young population in Vietnam, especially regions that have unique circumstances. Third, reporting sexual activities is sensitive in Vietnam, so our results might not accurately reflect attitudes and behaviors. Finally, our study had no data on the prevalence of youths and adolescents who belonged to the LGBTQA+ community in Vietnam, or exact information about the synthetic drugs that the participants used. Further studies could provide detailed data for each specific region of the country or specific gender groups and types of drugs used. However, the strength of our research is that we used the self-administered approach, therefore, our participants may have been more accurate in disclosing their sexual behaviors. In the reports of Survey Assessment of Vietnamese Youth (SAVY1-2003 and SAVY2-2008), there was only the general information about pubertal age, attitudes and practices of sexual behaviors, and reproductive health among young Vietnamese. Our study has provided more proper information about sexual behaviors among the youth which could contribute to more practical recommendations.

## 5. Conclusions

This study indicated that the awareness about condoms among the youth in Vietnam is significantly high; however, risky sexual behaviors remained common as well. Hence, future interventions should be implemented to focus on consistent condom use to prevent HIV/STIs and unintended pregnancy. In addition, sex-related education programs on the consequences of using alcohol and other stimulants as well as having multiple sex partners should be developed for reaching the youth at the locations where they often gather, such as schools. Early sexual health literacy may also help reduce unsafe sex.

## Figures and Tables

**Table 1:** Socioeconomic characteristics and risk behaviors.

	Individuals Reporting Sexual Experience				Total		*p*-Value
	Yes		No				
	n	%	n	%	n	%	
**Total**	882	73.5	318	26.5	1200	100	
**Gender**							
Male	480	84.81	86	15.19	566	47.2	0.88
Female	402	63.41	232	36.59	634	52.8	
**Living Area**							
Rural	249	333	87	138	336	28	<0.01
Urban	333	882	138	318	471	39.3	
Mountainous	300	249	93	87	393	32.7	
**Employment**							
Blue-collar worker	176	68.22	82	31.78	258	21.5	<0.01
Farmer	244	75.78	78	24.22	322	26.8	
High school student	47	41.96	65	58.04	112	9.3	
Undergraduate student	138	70.41	58	29.59	196	16.3	
White-collar worker	277	88.78	35	11.22	312	26.1	
**Age Group**							
16–18 years old	83	46.89	94	53.11	177	14.8	<0.01
19–24 years old	293	67.51	141	32.49	434	36.2	
25–30 years old	506	85.91	83	14.09	589	49	
**Smoking Tobacco**							
No	469	64.07	263	35.93	732	61	<0.01
Yes	413	88.25	55	11.75	468	39	
**Alcohol Drinking**							
No	164	47.26	183	52.74	347	28.9	<0.01
Yes	718	84.17	135	15.83	853	71.1	
**Other Substances Use (shisha, heroin…)**							
No	792	73.27	289	26.73	1081	90.1	0.33
Yes	90	75.63	29	24.37	119	9.9	

**Table 2:** Attitude and practice on sexual behaviors.

	n	%
**Want to Use Condom When Having Sex**	1200	67.0
**Ever had Sex in the Lifetime**		
Yes	882	73.5
No	318	26.5
**Ever had Unintended Pregnancy/Made Someone Pregnant**		
No	967	80.6
Yes	233	19.4
**Age at the 1^st^ Sexual Intercourse**		
Under 16	55	6.2
16–18 years old	260	29.5
Above 18 years old	567	64.3
**Number of Sex Partners**		
1–2 people	573	65.0
3–5 people	270	30.6
More than 5	39	4.4
**Ever Took Alcohol or other Stimulants before Having Sex**		
No	438	49.7
Yes	444	50.3
**Used a Condom at the Latest Sexual Intercourse**		
No	458	51.9
Yes	424	48.1

**Table 3:** Associated factors with attitude and practice on sexual behaviors.

Characteristics	Have Ever had Sex		Do not Want to Use Condoms		Do not Use Condoms		Unintended Pregnancy	
	OR	95% CI	OR	95% CI	OR	95% CI	OR	95% CI
**Occupation**								
Workers	ref		ref		ref		ref	
Farmers	0.63 **	0.44; 0.90						
Pupils	0.17 ***	0.10; 0.29	0.88	0.42; 1.82			0.30	0.07; 1.37
Students			0.29 ***	0.18; 0.48	0.17 ***	0.09; 0.31	0.35 ***	0.18; 0.69
White-collar workers			0.63 ***	0.45; 0.87			1.44 *	0.96; 2.16
**Living Area**								
Urban	ref		ref		ref		ref	
Rural			1.48 ***	1.11; 1.97			1.86 ***	1.16; 2.98
Mountainous							1.69 **	1.06; 2.69
**Age**								
16–18 years old	ref		ref		ref		ref	
19–24 years old			2.11 **	1.11; 4.03	3.33 ***	1.63; 6.77		
25–30 years old	2.24 ***	1.61; 3.10	3.07 ***	1.64; 5.77	1.85*	0.94; 3.64		
**Gender**								
Male	ref		ref		ref		ref	
Female	0.31 ***	0.22; 0.45	0.76 *	0.58; 1.02	1.46 **	1.05; 2.01	1.53 **	1.06; 2.20
**Have ever Drunk Alcohol**								
No	ref		ref		ref		ref	
Yes	4.50 ***	3.30; 6.13	0.67 **	0.48; 0.92	0.63 *	0.40; 1.00	0.61 **	0.39; 0.94
**Have ever Used other Substances**								
No	ref		ref		ref		ref	
Yes	1.52	0.91; 2.55			10.50 ***	5.23; 21.07		
**Have ever had Sex**								
Yes	ref		ref		ref		ref	
No			2.23 ***	1.61; 3.09				
**Had Sex for the First Time**								
Before 16	ref		ref		ref		ref	
At 16–18					0.15 ***	0.07; 0.33	0.51 ***	0.32; 0.81
After 18					0.50 *	0.23; 1.11		
**Number of Sex Partners**								
1–2 people	ref		ref		ref		ref	
3–5 people					2.18 ***	1.55; 3.07	1.33	0.93; 1.91
**Taking Alcohol or other Stimulants before having Sex**								
Yes	ref		ref		ref		ref	
No							1.46 **	1.03; 2.07
**Do not Want to Use Condoms**								
Yes	ref		ref		ref		ref	
No							2.85 ***	1.98; 4.10

*** *p* < 0.01, ** *p* < 0.05, * *p* < 0.1.
